# Antithrombin activity levels for predicting long-term outcomes in the early phase of isolated traumatic brain injury

**DOI:** 10.3389/fimmu.2022.981826

**Published:** 2022-09-29

**Authors:** Masaki Takahashi, Takeshi Wada, Ryuta Nakae, Yu Fujiki, Takahiro Kanaya, Yasuhiro Takayama, Go Suzuki, Yasutaka Naoe, Shoji Yokobori

**Affiliations:** ^1^ Division of Acute and Critical Care Medicine, Department of Anesthesiology and Critical Care Medicine, Hokkaido University Faculty of Medicine, Sapporo, Japan; ^2^ Department of Emergency and Critical Care Medicine, Nippon Medical School Hospital, Tokyo, Japan; ^3^ Emergency and Critical Care Center, Kawaguchi Municipal Medical Center, Saitama, Japan

**Keywords:** antithrombin, traumatic brain injury, trauma-induced coagulopathy, disseminated intravascular coagulation, long-term outcome

## Abstract

Coagulopathy management is an important strategy for preventing secondary brain damage in patients with traumatic brain injury (TBI). Antithrombin (AT) is a natural anticoagulant that controls coagulation and inflammation pathways. However, the significance of AT activity levels for outcomes in patients with trauma remains unclear. This study aimed to investigate the relationship between AT activity levels and long-term outcomes in patients with TBI; this was a sub-analysis of a prior study that collected blood samples of trauma patients prospectively in a tertiary care center in Kawaguchi City, Japan. We included patients with isolated TBI (iTBI) aged ≥16 years admitted directly to our hospital within 1 h after injury between April 2018 and March 2021. General coagulofibrinolytic and specific molecular biomarkers, including AT, were measured at 1, 3, 6, 12, and 24 h after injury. We analyzed changes in the AT activity levels during the study period and the impact of the AT activity levels on long-term outcomes, the Glasgow Outcome Scale-Extended (GOSE), 6 months after injury. 49 patients were included in this study; 24 had good neurological outcomes (GOSE 6–8), and 25 had poor neurological outcomes (GOSE 1–5). Low AT activity levels were shown within 1 h after injury in patients in the poor GOSE group; this was associated with poor outcomes. Furthermore, AT activity levels 1 h after injury had a strong predictive value for long-term outcomes (area under the receiver operating characteristic curve of 0.871; 95% CI: 0.747–0.994). Multivariate logistic regression analysis with various biomarkers showed that AT was an independent factor of long-term outcome (adjusted odds ratio: 0.873; 95% CI: 0.765–0.996; p=0.043). Another multivariate analysis with severity scores showed that low AT activity levels were associated with poor outcomes (adjusted odds ratio: 0.909; 95% CI: 0.822–1.010; p=0.063). We demonstrated that the AT activity level soon after injury could be a predictor of long-term neurological prognosis in patients with iTBI.

## Introduction

Approximately sixty-nine million persons worldwide suffer a traumatic brain injury (TBI) yearly (1). Despite advances in neuroprotective care and surgery, TBI remains a leading cause of death and disability (2), especially in young populations (3). Head injury can cause various types of damage, such as fracture of the skull, cerebral laceration or contusion, diffuse axonal injury, and intracranial hemorrhage (ICH). Although the primary injury (the direct mechanical brain damage) is inevitable and untreatable (4), many efforts have been made in clinical settings to minimize the secondary injury (delayed non-mechanical damage) (5, 6).

Coagulopathy management is one of the strategies to protect the brain after an injury (7). Dynamic coagulofibrinolytic changes, called trauma-induced coagulopathy (TIC), are observed soon after injury in patients with trauma, including TBI (8). Although many studies have reported that TIC is associated with poor outcomes (9, 10), its mechanism has not been established and remains controversial. A recent study revealed that patients with TIC showed hypercoagulation, consumption coagulopathy, and hyperfibrinolysis, which are equivalent to disseminated intravascular coagulation (DIC) with hyperfibrinolysis (11). The basic essence of DIC is the dysregulation of inflammatory and coagulofibrinolytic responses to insults, and damage-associated molecular patterns (DAMPs) play an important role in DIC. In trauma, DAMPs such as histones are released from injured cells. They activate innate immune responses, leading to inflammation and coagulation activation, which are essentially physiological responses for hemostasis and tissue repair. However, if the trauma is sufficiently severe, these responses change from physiological to pathological conditions, resulting in DIC development (12).

Antithrombin (AT), a natural anticoagulant, plays an important role in the coagulation pathway by showing 80% inhibitory activity against thrombin (13). Low AT activity levels have been associated with multiple organ failure syndrome and high mortality in patients with septic DIC (14). However, the significance of AT activity levels for outcomes in trauma patients has not been investigated in detail. Dunbar et al. reported that low AT activity levels promoted thrombin generation in trauma patients (15), which could cause anticoagulation dysfunction followed by additional thrombin generation.

We hypothesized that AT is an important factor affecting prognosis, especially long-term outcomes, in patients with TBI. This study aimed to assess AT activity levels as a predictor of long-term neurological outcomes in patients with isolated TBI (iTBI).

## Materials and methods

### Study design, ethical approval, and patient selection

This study is a sub-analysis using samples collected for a prior study that analyzed the time course of coagulation, fibrinolysis, and fibrinolytic shutdown in trauma patients. Blood samples were collected in a tertiary care center in Kawaguchi City, Japan. The study was approved by relevant institutional review boards (Hokkaido University Hospital: #021-0185, March 11, 2022, Nippon Medical School: #M-2021-025, February 16, 2022, and Kawaguchi Municipal Medical Center: #2018-27, November 20, 2018), and performed in accordance with the ethical principles of the Declaration of Helsinki. We performed a retrospective observational study with the collected data in this study. This study included patients with iTBI aged ≥16 years, admitted directly to our hospital within 1 h after injury between April 2018 and March 2021. We defined iTBI as an Abbreviated Injury Scale (AIS) score of ≥3 for the head and ≤2 for other body parts. We excluded patients with any life-threatening complications or any treatment restrictions because of advance directives.

### Data collection and measurements

Demographic and clinical information was collected, including age, sex, Glasgow Coma Scale (GCS) upon admission, Injury Severity Score (ISS), AIS of each body area, the volume of blood transfusion, Acute Physiology and Chronic Health Evaluation II (APACHE II) score, type of intracranial injury, and long-term neurological prognosis. The long-term prognosis was evaluated using the Glasgow Outcome Scale-Extended (GOSE) 6 months after injury, and good and poor neurological outcomes were defined as GOSE ≥ 6 and ≤ 5, respectively. Blood samples were collected at 1, 3, 6, 12, and 24 h after injury. In addition to general coagulofibrinolytic biomarkers, specific molecular biomarkers were assessed, including (i) thrombin-antithrombin complex (TAT) (a marker of thrombin generation) (STACIA CLEIA TAT^®^, LSI Medience Corp., Tokyo, Japan), (ii) AT (a marker of anti-thrombin) (Revohem AT^®^, Sysmex Corp., Kobe, Japan), (iii) plasmin-alpha 2-plasmin inhibitor complex (PIC) (a marker of plasmin generation) (LPIA-ACE PPI II^®^, LSI Medience Corp., Tokyo, Japan), (iv) alpha 2-plasmin inhibitor (α_2_-PI) (a marker of inhibition of plasmin) (Testzym S APL^®^, Sekisui Medical Corp., Tokyo, Japan), (v) plasminogen (the zymogen form of plasmin) (Testzym S PLG^®^, Sekisui Medical Corp., Tokyo, Japan), and (vi) plasminogen activator inhibitor-1 (PAI-1) (a marker of inhibition of fibrinolysis) (LPIA-tPAI test^®^, LSI Medience Corp., Tokyo, Japan).

### Statistical analyses

All variables are presented as median (interquartile range) or numbers (percentage). Missing values were removed from the analysis, and intergroup comparisons were performed using the Mann-Whitney U and chi-square test for continuous and categorical variables, respectively. The changes in variables were analyzed using the Friedman test (repeated measures). The predictive values of the biomarkers for long-term prognosis were assessed using the area under the receiver operating characteristic (ROC) curve (AUC) and multivariate logistic regression analysis. The optimal cut-off points were calculated using the Youden index from the ROC curves. Differences with a two-tailed p<0.05 were considered statistically significant. All statistical analyses were performed using R software version 4.1.2 (R Core Team. R Foundation for Statistical Computing, Vienna, Austria).

## Results

### Baseline characteristics, severity, and outcome

This study included 49 patients; 24 had good neurological outcomes, and 25 had poor neurological outcomes. The baseline characteristics, severity, and outcome of the study population are shown in **Table 1**. Among the included patients, the median age was 66 years; thirty-nine (79.6%) patients survived to discharge, and twenty-four (49.0%) patients had long-term favorable neurological outcomes. Patients in the poor GOSE group (GOSE 1–5) showed higher median age and worse severity scores, such as the GCS motor score, AIS of the head, ISS, and APACHE II score, than those in the good GOSE group (GOSE 6–8).

**Table 1:** The baseline characteristics, severity, and outcome of the study population.

	All patients	Good GOSE group	Poor GOSE group	
	n = 49	n = 24	n = 25	p-value
Sex, male	29 (59.2)	13 (54.2)	16 (64.0)	0.567
Age, years	66 [41–80]	44.5 [31.5–67.75]	80.0 [62.0–82.0]	<0.001
Mechanism of trauma				0.087
Traffic accident	27 (55.1)	17 (70.8)	10 (40.8)	
Fall	11 (22.4)	4 (16.7)	7 (28.0)	
Others	11 (22.4)	3 (12.5)	8 (32.0)	
GCS at arrival	12 [6–14]	13 [12–15]	6 [5–13]	0.001
Eye	3 [1–4]	3 [3–4]	1 [1–3]	0.014
Verbal	3 [1–4]	4 [3–5]	1 [1–4]	0.001
Motor	5 [4–6]	6 [5.75–6]	4 [3–5]	<0.001
Pupil dilation, yes (at arrival)	17 (34.7)	9 (37.5)	8 (32.0)	0.769
Anisocoria, yes (at arrival)	14 (28.6)	4 (16.7)	10 (40.0)	0.114
Types of injury				
Skull fracture	32 (65.3)	15 (62.5)	17 (68.0)	0.769
TSAH	46 (93.9)	22 (91.7)	24 (96.0)	0.609
ASDH	40 (81.6)	13 (54.3)	24 (96.0)	<0.001
AEDH	7 (14.3)	5 (20.8)	2 (8.0)	0.247
Brain contusion	41 (83.7)	18 (75.0)	23 (92.0)	0.138
AIS				
Head	5 [4–5]	4 [3–5]	5 [5–5]	<0.001
Face	0 [0–0]	0 [0–0]	0 [0–0]	0.071
Chest	0 [0–0]	0 [0–0]	0 [0–0]	0.307
Abdomen	0 [0–0]	0 [0–0]	0 [0–0]	0.307
Extremities	0 [0–0]	0 [0–0]	0 [0–0]	0.204
External	0 [0–0]	0 [0–0]	0 [0–0]	NA
ISS	25 [16–25]	16.5 [13.75–25]	25 [25–25]	0.002
Transfusion				
RBC (unit)	0 [0–4]	0 [0–0]	2 [0–10]	0.001
FFP (unit)	0 [0–6]	0 [0–0]	0 [0–10]	0.009
Operation for TBI	29 (59.2)	8 (33.3)	21 (84.0)	<0.001
APACHE II	14.00 [9.75–20.25]	10.00 [7.25–13.00]	20.0 [15.00–23.00]	<0.001
GOSE at 6 months	5 [3-7]	7 [7–8]	3 [1–4]	<0.001
Mortality	10 (20.4)	0 (0)	10 (40.0)	0.001

All data are presented as median [interquartile range] or N (%).

GOSE, Glasgow Outcome Scale-Extended; GCS, Glasgow Coma Scale; TSAH, traumatic subarachnoid hemorrhage;

ASDH, acute subdural hematoma; AEDH, acute extradural hematoma; AIS, Abbreviated Injury Scale; ISS, Injury Severity Score; RBC, red blood cell;

FFP, fresh frozen plasma; TBI, traumatic brain injury; APACHE II, Acute Physiology and Chronic Health Evaluation II.

### Comparison of coagulofibrinolytic biomarkers between good and poor GOSE groups

**Figure 1:**
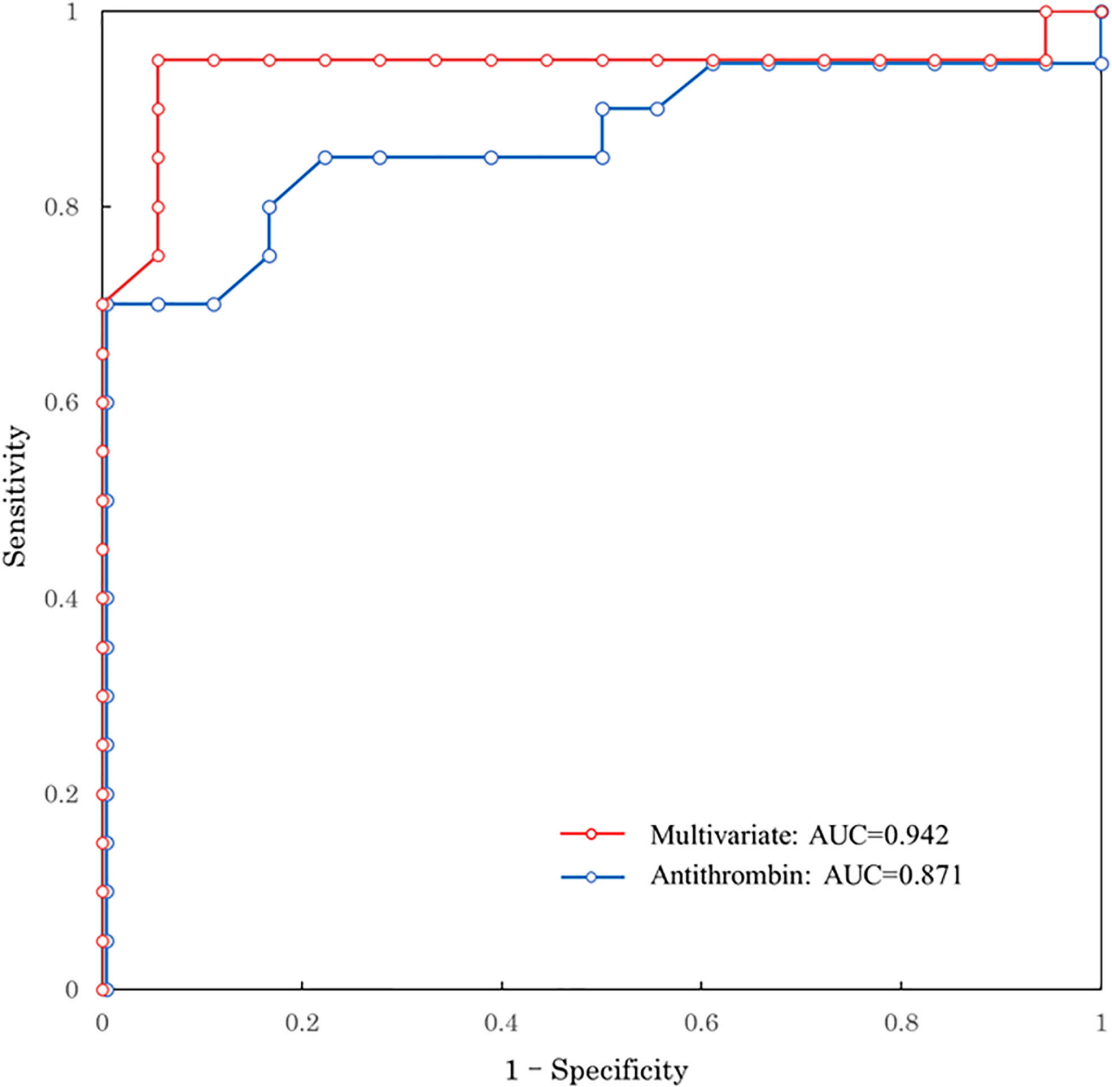
ROC curve predicting long-term neurological outcome. Red line: multivariate logistic regression model with age, GCS, ISS, and antithrombin 1 h after injury Blue line: univariate of antithrombin 1h after injury. ROC, receiver operating characteristic; AUC, area under the ROC curve; GCS, Glasgow Coma Scale; ISS, Injury Severity Score.

**Table 2** shows the biomarkers measured in the good and poor GOSE groups 1 h after injury. As mentioned above, patients in the poor GOSE group suffered more severe injury than those in the good GOSE group; furthermore, all biomarkers, except for fibrinogen, TAT, plasminogen, and PAI-1, showed a significant difference in their levels between the two groups 1 h after injury. We further observed similar results at other time points (**Supplementary Tables S1–S4**). **Table 2** also shows the univariate predictive values of biomarkers for long-term neurological outcomes. AT shows the highest AUC of 0.871 (95% confidence interval [CI]: 0.747–0.994). The ROC curve of AT is shown in **Figure 1**. The optimal cut-off value of AT activity level for long-term neurological prognosis was 80%. The sensitivity and specificity for poor outcomes were 70.0% and 100%, respectively.

**Table 2:** Biomarkers of good and poor GOSE groups 1 h after injury.

	Good GOSE group	Poor GOSE group		
	n = 24	n = 25	p-value	AUC (95% CI)
Platelet count (×10^4^/dL)	22.9 [20.1–27.0]	17.7 [12.4–20.4]	0.001	0.766 (0.626–0.905)
PT-INR	1.02 [0.98–1.06]	1.08 [1.01–1.17]	0.028	0.683 (0.533–0.834)
APTT (sec)	25.2 [24.1–27.3]	29.4 [26.8–33.2]	<0.001	0.800 (0.674–0.926)
Fibrinogen (mg/dL)	214 [187–259]	195 [138–270]	0.250	0.596 (0.427–0.765)
FDP (μg/mL)	56.9 [21.3–107.9]	255.4 [88.1–533.1]	<0.001	0.817 (0.696–0.937)
D-dimer (μg/mL)	16.6 [6.7–28.7]	53.2 [28.7–139.1]	<0.001	0.808 (0.685–0.932)
TAT (mg/mL)	120.0 [60.6–120.0]	120.0 [120.0–120.0]	0.111	0.607 (0.477–0.737)
Plasminogen (%)	90.0 [81.0–101.0]	84.0 [72.8–94.0]	0.118	0.633 (0.467–0.799)
α_2_-PI (%)	96.0 [87.0–103.0]	73.5 [58.5–91.3]	0.001	0.789 (0.650–0.927)
PIC (μg/mL)	4.4 [2.2–9.6]	17.0 [9.3–31.8]	<0.001	0.821 (0.700–0.941)
PAI-1 (ng/mL)	22.0 [11.5–23.5]	23.0 [15.3–48.5]	0.169	0.617 (0.452–0.782)
Antithrombin (%)	93.5 [88.5–100.5]	77.0 [69.0–84.3]	<0.001	0.871 (0.747–0.994)

All data are presented as median [interquartile range].

AUCs show predictive accuracy for long-term neurological outcomes of the various univariate variables.

GOSE, Glasgow Outcome Scale-Extended; AUC, area under the receiver operating characteristic curve;

PT-INR, prothrombin time international normalized ratio; APTT, activated partial thromboplastin time; FDP, fibrin/fibrinogen degradation products;

TAT, thrombin-antithrombin complex; α_2_-PI, alpha 2-plasmin inhibitor; PIC, plasmin/alpha 2-antiplasmin complex; PAI-1, plasminogen activator inhibitor-1.

### Multivariate logistic regression for long-term outcomes

Results for unadjusted (univariate) and age-adjusted logistic regression analyses are shown in **Table 3**. After age adjustment, prothrombin time international normalized ratio, activated partial thromboplastin time, D-dimer, α_2_-PI, PIC, PAI-1, and AT remained statistically significant factors. Multivariate logistic regression analysis was performed to identify the independent factors for long-term outcomes. Limited variables were entered into the model because of the small sample size. In addition to age, we selected the key markers of coagulation and fibrinolysis as variables: markers of thrombin generation (TAT), anti-thrombin (AT), plasmin generation (PIC), inhibition of fibrinolysis (PAI-1), and platelet count. **Table 3** shows that only AT was an independent factor (adjusted odds ratio, 0.873; 95% CI: 0.765–0.996; p=0.043). To assess the relationship between AT activity levels and other severity scores, we performed another multivariate logistic regression analysis with age, GCS, ISS, and AT. As shown in **Table 4**, low AT activity level was associated with poor neurological outcomes, but was not statistically significant in this analysis (adjusted odds ratio: 0.909; 95% CI: 0.822–1.010; p=0.063). Using this logistic model, a multivariate ROC curve was depicted in **Figure 1**. The multivariate model showed the AUC of 0.942 (95% CI: 0.847–1.000).

**Table 3:** Univariate, age-adjusted, and multivariate logistic regression analyses of biomarkers 1 h after injury.

Variable	Univariate		Age-adjusted		Multivariate	
	OR (95% CI)	p-value	OR (95% CI)	p-value	OR (95% CI)	p-value
Age (year)	1.060 (1.020–1.100)	0.001			1.090 (0.998–1.190)	0.054
Platelet count (×10^4^/dL)	0.864 (0.777–0.926)	0.007	0.919 (0.826–1.020)	0.122	0.992 (0.777–1.270)	0.945
PT-INR (OR per 0.01 unit)	1.080 (1.000–1.160)	0.039	1.120 (1.020–1.230)	0.020		
APTT (sec)	1.360 (1.090–1.680)	0.005	1.330 (1.050–1.700)	0.020		
Fibrinogen (mg/dL)	0.997 (0.989–1.000)	0.430	0.996 (0.987–1.010	0.415		
FDP (μg/mL)	1.010 (1.000–1.020)	0.004	1.010 (1.000–1.010)	0.018		
D-dimer (μg/mL)	1.030 (1.010–1.060)	0.011	1.030 (1.000–1.060)	0.039		
TAT (mg/mL)	1.010 (0.996–1.030)	0.137	1.010 (0.999–1.030)	0.237	1.020 (0.987–1.060)	0.212
Plasminogen (%)	0.970 (0.935–1.010)	0.105	1.050 (1.020–1.090)	0.380		
α_2_-PI (%)	0.936 (0.896–0.978)	0.003	0.952 (0.911–0.995)	0.029		
PIC (μg/mL)	1.110 (1.030–1.200)	0.007	1.080 (1.000–1.170)	0.049	0.937 (0.802–1.090)	0.409
PAI-1 (ng/mL)	1.030 (0.997–1.050)	0.080	1.040 (1.000–1.080)	0.038	1.040 (0.993–1.100)	0.094
Antithrombin (%)	0.887 (0.818–0.962)	0.004	0.892 (0.815–0.976)	0.013	0.873 (0.765–0.996)	0.043

OR shows the ratio of the odds of an increase in the predictor by a defined unit.

PT-INR, prothrombin time international normalized ratio; APTT, activated partial thromboplastin time; FDP, fibrin/fibrinogen degradation products;

TAT, thrombin-antithrombin complex; α_2_-PI, alpha 2-plasmin inhibitor; PIC, plasmin/alpha 2-antiplasmin complex; PAI-1, plasminogen activator inhibitor-1.

**Table 4:** Univariate and multivariate logistic regression analyses of antithrombin 1 h after injury and other severity scores.

Variable	Univariate		Multivariate	
	OR (95% CI)	p-value	OR (95% CI)	p-value
Age (year)	1.060 (1.020–1.100)	0.001	1.070 (1.000–1.140)	0.037
GCS at arrival	0.744 (0.627–0.883)	<0.001	0.756 (0.560–1.020)	0.067
ISS	1.200 (1.060–1.350)	0.005	1.020 (0.858–1.220)	0.791
Antithrombin (%)	0.887 (0.818–0.962)	0.004	0.909 (0.822–1.010)	0.063

OR shows the ratio of the odds of an increase in the predictor by a defined unit.

GCS, Glasgow Coma Scale; ISS, Injury Severity Score.

### Time course of AT activity levels change

**Figure 2:**
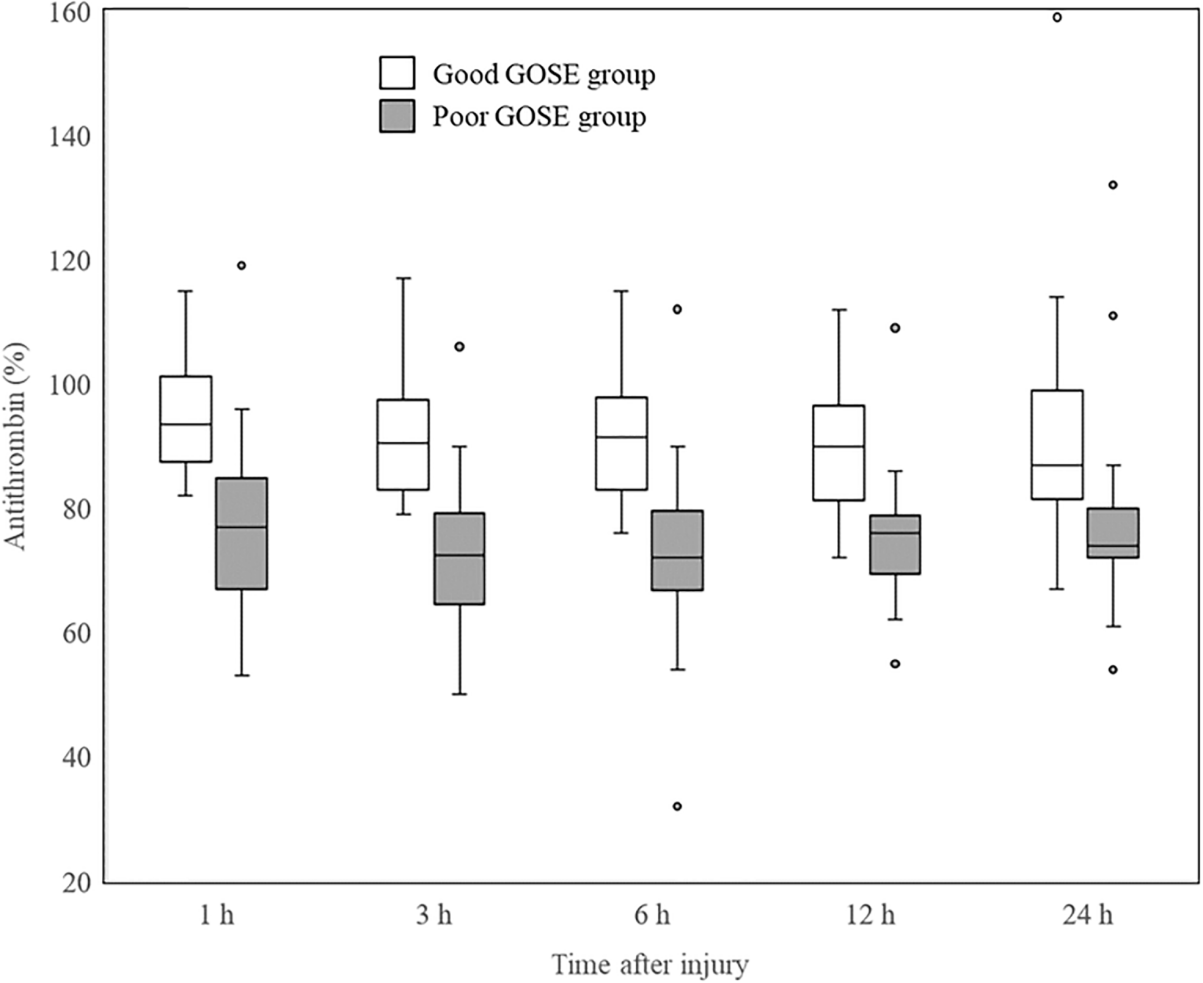
Changes in AT activity levels of each GOSE group after injury. Significant differences between good (white box) and poor (gray box) GOSE groups at all the time points are shown in AT activity levels. Neither group showed a significant change in AT activity levels after injury (good GOSE group, Friedman test, χ^2^ = 3.59, df = 4, p = 0.464; poor GOSE group, Friedman test, χ^2^ = 2.83, df = 4, p = 0.587). Horizontal bars, boxes, whiskers, and dots represent the median, interquartile range, a distance of 1.5 times the interquartile range, and outliers, respectively. AT, antithrombin; GOSE, Glasgow Outcome Scale-Extended.

The time course of the change in AT activity levels was analyzed in all included patients, with no significant changes over the study period (Friedman test, χ^2^ = 3.95, df=4, p=0.413). The changes in AT activity levels for each GOSE group are shown in **Figure 2**. Although the poor GOSE group had lower AT activity levels than the good GOSE group at all time points, neither group showed a significant change in AT activity levels after injury (good GOSE group, Friedman test, χ^2^ = 3.59, df=4, p=0.464; poor GOSE group, Friedman test, χ^2^ = 2.83, df=4, p=0.587).

## Discussion

This study investigated the association between AT activity levels and long-term outcomes in patients with iTBI. We demonstrated that the AT activity level, among various biomarkers, is an independent predictor of long-term neurological prognosis. Patients in the poor prognosis group showed lower AT activity levels than those in the good prognosis group soon after injury. Furthermore, AT activity levels 1 h after an injury had a strong predictive value for long-term neurological outcomes. We also found that AT activity levels did not change significantly after the first hour after injury. This study was designed to collect blood samples from the injury time, which could reduce the bias such as differences in transport time between patients. Therefore, we investigated the time course of AT activity levels in patients with iTBI in detail.

We demonstrated that low AT activity levels were strongly associated with poor long-term prognosis in patients with iTBI. However, we could not demonstrate a causal relationship between AT activity levels and outcomes. Several possible reasons for the relationship between AT activity levels and outcomes have been considered. First, thrombin generation promoted by decreased AT could lead to consumption coagulopathy (16), resulting in ICH progression and poor outcomes. However, ICH progression after TBI could be caused by various factors, such as hyperfibrinolysis, platelet dysfunction, inflammation, and endothelial activation (17). Hence, it remains unclear how much AT activity levels can affect ICH progression. Second, decreased AT activity levels in trauma patients are associated with DIC (10, 18), which causes poor long-term prognosis. There are two DIC phenotypes: the fibrinolytic and thrombotic (19, 20).

Conversely, TIC has been reported to have multiple coagulopathy phase in trauma patients. Severe bleeding is observed in the early phase of trauma because of consumption coagulopathy caused by activated coagulation and hyperfibrinolysis, which is equivalent to the fibrinolytic DIC phenotype. In the late phase, fibrinolysis suppression increases the risk of thrombosis and multiple organ dysfunction syndrome (MODS) like the thrombotic DIC phenotype (8). The incidence of cerebral infarction also increases during the late phase of trauma (21). Nonetheless, ICH or cerebral infarction leads to worse neurological outcomes. Further investigation into the cause of poor outcomes is needed to confirm the significance of AT activity levels.

Although previous studies have showed that fibrinolytic markers were predictors of the progression of intracranial hemorrhage (22, 23) in patients with TBI, we did not find PIC, a fibrinolytic marker, to be associated with prognosis in the multivariate analysis. There were several possible reasons for this finding. First, although previous studies (22, 23) analyzed D-dimer as a fibrinolytic marker, we evaluated PIC in the multivariate analysis. Although D-dimer is a marker that reflects activation of both coagulation and fibrinolysis (24), PIC reflects the degree of fibrinolysis more accurately (25); therefore, we selected PIC as a fibrinolytic marker. Second, our multivariate model incorporated AT as a variable, which differs from previous studies. Third, we evaluated the long-term outcomes of patients. Deaths due to expansion of intracranial hemorrhage occur in the acute phase. However, after the subacute phase, patients with TBI could die due to MODS, which is caused by a thrombotic DIC phenotype, due to low AT activity. Fibrinolytic markers could predict the progression of intracranial hemorrhage and short-term outcomes. However, AT could affect both the progression of hemorrhage and MODS; AT could be a predictor of short and long-term prognosis, including early deaths.

Thrombin and AT play important roles in coagulation and inflammation (26, 27), and low AT activity levels could be a high risk for dysregulated coagulation and inflammation. The activation of coagulation and inflammation in patients with trauma is induced by trauma insult, which are essential reactions by the innate immune system for hemostasis and wound healing (28). However, excessive immune responses could lead to dysregulated thrombosis formation and inflammation, namely DIC, contributing to the development of MODS and systemic inflammatory response syndrome. Considering the roles of AT in coagulation and inflammation, AT is one of the essential factors in innate immunity.

The mechanism of AT activity depletion in trauma has not yet been fully elucidated. One possible mechanism is increased consumption of AT (18). In the early phase of trauma, including TBI, DAMPs released into circulation lead to a hypercoagulable state and thrombin generation (29, 30). AT, a natural anticoagulant, neutralizes increased thrombin, resulting in AT consumption to form TAT (31). Thus, lower AT activity levels could imply a hypercoagulable state enhanced by trauma insult, which equals a hyperinflammatory state. In this study, low AT activity levels seemed to be associated with high TAT levels; however, measured TAT levels showed upper limits in many cases, complicating the quantitative evaluation.

Previous studies have reported that AT activity levels decreased due to extravascular leakage in patients with DIC (32) and trauma (33). These reports showed that AT activity levels strongly correlated with serum albumin levels, but not TAT levels. However, to our knowledge, no study has examined patients with TBI. Critically ill patients with sepsis suffer damage to endothelial cells and glycocalyx (endotheliopathy), which causes the breakdown of tight junctions with extravascular leakage of albumin or AT (34). Syndecan-1, one of the biomarkers of endotheliopathy, is a predictor of mortality (35) and sepsis (36) in patients with trauma. Conversely, one previous study indicated that patients with iTBI did not have syndecan-1 levels as high as those in polytrauma patients with TBI (37). Based on the above, the risk of extravascular leakage may not be high in patients with iTBI. Hence, we considered that the main mechanism of AT activity depletion was not extravascular leakage and did not evaluate it in this study. However, because of the interaction between coagulation and inflammation (38), it is reasonable to assume that AT activity depletion in trauma is related to both increased consumption and extravascular leakage.

Most patients in this study did not require massive transfusion or fluid infusion because patients with iTBI rarely experience hemorrhagic shock. Therefore, it is considered that AT activity levels could decrease without plasma dilution or blood loss. In this study, we found that AT activity levels did not significantly change after admission in patients with iTBI, even among those whose AT activity levels had decreased 1 h after injury (**Figure 2**). In other words, the period during which AT activity levels often decreases is within 1 h of injury. The reason for these time-associated AT changes is unclear. One possible mechanism considered is that the activation of coagulation reaches a peak 1 h after trauma injury (39); therefore, AT consumption also peaks simultaneously. In addition, the AT activity levels in some patients in this study might have been low even before injury.

Importantly, we did not demonstrate the necessity of AT administration to patients with iTBI. A previous small study investigated the efficacy of AT administration in patients with TBI and did not find any significant influence on prognosis (40). However, a recent study reported that AT could reduce neuromicrovascular permeability in mouse models of TBI (41). Further study is required to clarify the significance of AT activity levels and administration in patients with TBI.

This study had several limitations. First, this was a single-center observational study; data with missing values were collected prospectively and analyzed retrospectively. Second, this study included a relatively small number of patients for the multivariate logistic regression analysis. Therefore, the results were partially supported by the statistical analysis. Third, some variables, such as fibrin/fibrinogen degradation products, D-dimer, and TAT, had the upper limit of the measuring range, resulting in a bias during the quantitative evaluation. Fourth, we did not assess the degree of inflammation or extravascular leakage, and the mechanism of AT depletion remains unclear.

## Conclusion

The AT activity level is an independent predictor of long-term neurological prognosis in patients with iTBI; lower AT activity levels soon after injury are associated with a poor prognosis. Large-scale studies are needed to clarify the significance of AT in patients with TBI.

## Data availability statement

The raw data supporting the conclusions of this article will be made available by the authors, without undue reservation.

## Ethics statement

The studies involving human participants were reviewed and approved by Hokkaido University Hospital Nippon Medical School Kawaguchi Municipal Medical Center. Written informed consent to participate in this study was provided by the participants’ legal guardian/next of kin.

## Author contributions

MT analyzed the study results, interpreted the data, and drafted the manuscript. TW oversaw the entire research and contributed to the research concept, the analysis, and interpretation of the data, and was responsible for drafting, editing, and submitting the manuscript. RN, YF, TK, GS, and YN collected the data. SY supervised the study. All authors have read and approved the final manuscript.

## Funding

This study was supported in part by JSPS KAKENHI Grant Number 19K18367. The funders had no role in the execution of this study or interpretation of the results.

## Acknowledgments

We would like to thank Editage (https://online.editage.jp/) for English language editing.

## Conflict of interest

The authors declare that the research was conducted in the absence of any commercial or financial relationships that could be construed as a potential conflict of interest.

## Publisher’s note

All claims expressed in this article are solely those of the authors and do not necessarily represent those of their affiliated organizations, or those of the publisher, the editors and the reviewers. Any product that may be evaluated in this article, or claim that may be made by its manufacturer, is not guaranteed or endorsed by the publisher.
